# Greek Raw Honey from Pindos Mountain Improves Redox Homeostasis of RAW264.7 Macrophages

**DOI:** 10.3390/ijms26072868

**Published:** 2025-03-21

**Authors:** Anastasia Patouna, Fotis Tekos, Myrto Charouli, Periklis Vardakas, Demetrios Kouretas

**Affiliations:** Department of Biochemistry and Biotechnology, School of Health Sciences, University of Thessaly, 41500 Larissa, Greece; anpatouna@uth.gr (A.P.); ftekos@uth.gr (F.T.); mcharouli@uth.gr (M.C.); pevardakas@uth.gr (P.V.)

**Keywords:** oxidative stress, antioxidants, redox homeostasis, macrophages, honey

## Abstract

Honey is a complex mixture of various compounds that possesses strong biological properties, among which is its antioxidant activity. It is worth mentioning that the botanical origin and the phytochemical composition are crucial parameters that determine the bioactive profile of honey. Oxidative stress is a biological phenomenon implicated into the pathogenesis of various diseases. Hence, the multifaceted evaluation of the redox-related effects of natural products, rich in bioactive compounds, may lead to the growth of putative strategies for the attenuation of oxidative stress and the prevention of such pathophysiological conditions. Within this context, the aim of the present study was to assess the biological activities of six Greek raw honey samples from Pindos Mountain in vitro, by examining their ability to cause redox alterations in RAW264.7 macrophages. For that purpose, we evaluated a panel of markers associated with antioxidant defense and oxidative damage. According to our findings, most honey samples had positive impacts on cellular redox homeostasis, as indicated by the enhancement of antioxidant defense mechanisms and the protection against oxidative damage to lipids and proteins. Conclusively, this study highlights the Greek raw honey samples potent antioxidant capacity, confirming their promising role in improving redox homeostasis.

## 1. Introduction

Honey is the most important product of honeybees, owing to its substantial quantity and relevance to human nutrition [1]. It is a highly nutritious, complex mixture comprising about 200 constituents, primarily sugars, water, and minor compounds such as phenolic and volatile, minerals, proteins, free amino acids, organic acids, enzymes, and vitamins [2]. Some honey physicochemical properties, such as viscosity and crystallization, depending on its sugar levels [3]. Furthermore, honey contains bioactive compounds with strong biological properties, such as chrysin, quercetin, and kaempferol [4,5,6]; for this reason, it is used in traditional medicine, exhibiting significant antioxidant, antibacterial, anti-inflammatory, and wound healing activities [7].

Honey is highly diversified based on the geographical location of the hive, a characteristic attributed to the varying climatic conditions and, hence, to the biodiversity that defines each region [8,9,10,11,12]. The Greek flora includes roughly 1100 endemic species, constituting the main factor of honey’s high quality [13,14]. Greek honey is notable for both its organoleptic properties [15] and biological activities [16,17], representing a key aspect of the country’s economy. According to the European Union (EU), Greece is one of the leading honey producers, accounting for 9.5% of EU production in 2022, also ranking first in terms of the ratio of hives per beekeeper (99 hives/beekeeper) [18].

In recent years, scientific interest has focused on dietary bioactive compounds for the purpose of developing putative strategies to prevent chronic diseases. The main rationale behind such investigations is their well-established health-promoting properties, non-toxicity, and cost-effectiveness. Redox homeostasis, referring to the balance between prooxidants and antioxidants within the biological systems, is crucial for maintaining the normal cellular function, signaling, and defense [19]. Critical amounts of reactive oxygen species (ROS) are essential to various physiological processes; however, their excessive accumulation disrupts redox homeostasis, leading to disturbances of redox signaling and molecular damage, a biological phenomenon described as oxidative stress [20,21]. Oxidative stress causes severe damage to DNA, lipids, and proteins in the cellular environment [22,23,24,25]. It is worth noting that oxidative stress plays a pivotal role in the initiation and progression of several pathological conditions, such as cardiovascular and autoimmune diseases, diabetes, cancer, and neurological disorders [26,27,28,29,30]. Previous studies have emphasized on the capacity of dietary bioactive compounds, such as polyphenols, to ameliorate oxidative stress and contribute to health maintenance [31,32,33,34].

As previously mentioned, honey is not only an important commercial commodity in Greece but also a high-quality product. The analysis of honey samples from different regions of Greece has revealed a wide spectrum of phenolic compounds, such as quercetin, gallic acid, ferulic acid, and chlorogenic acid. The concentration of these compounds varies according to both the beehive location and the honey type [35]. Several studies have focused on the bioactivity of specific compounds in honey, primarily due to its complex composition. However, a key limitation of this approach is the lack of knowledge regarding the potential synergistic interactions of these compounds. In this context, the purpose of the present study was to evaluate the bioactive properties of 6 raw honey samples, originated from Pindos Mountain range, Greece, by focusing on their impacts on the redox homeostasis of RAW264.7 macrophages. This research aims to identify the antioxidant properties of Greek honey samples, placing special emphasis on the different responses considering honey type.

## 2. Results

### 2.1. Determination of Total Phenolic Content (TPC)

Based on our results (Table 1), the Forest honey sample exhibited the higher TPC, i.e., 129.4 mg GAE/100 g honey, followed by the Oak honey sample, i.e., 124 mg GAE/100 g honey, and the Forest with oak honeydew honey sample, i.e., 115.4 mg GAE/100 g honey. Considering the rest of the honey samples, TPC was consistent among them.

### 2.2. Determination of the Effects on Cell Viability

**Figure 1:**
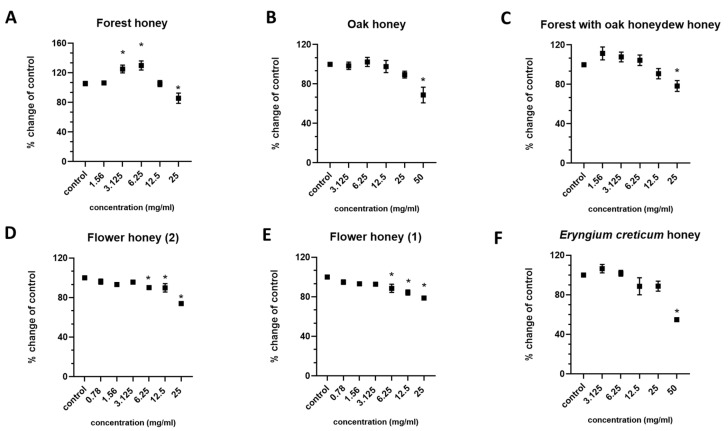
The cytotoxic effects of various concentrations (0.78 mg/mL–50 mg/mL) of (**A**) Forest, (**B**) Oak, (**C**) Forest with oak honeydew, (**D**) Flower (2), (**E**) Flower (1), and (**F**) Eryngium creticum honey samples on the cell viability of RAW264.7 macrophages. *: *p* < 0.05, statistically significant difference as compared to the control.

The honey samples were administrated at various concentrations (0.78 mg/mL–50 mg/mL) to RAW264.7 macrophages for the purpose of assessing the cytotoxic effects on cell viability. According to our results (Figure 1), the Flower honey samples (Figure 1D,E) exhibited the lowest cytotoxic threshold, i.e., 6.25 mg/mL. The Forest (Figure 1A) and Forest with oak honeydew (Figure 1C) honey samples showed a cytotoxic threshold of 25 mg/mL, whereas the Oak (Figure 1B) and *Eryngium creticum* (Figure 1F) honey samples showed the highest cytotoxic threshold, i.e., 50 mg/mL.

### 2.3. Determination of the Effects on Intracellular Reduced Glutathione (GSH) and Reactive Oxygen Species (ROS) Levels

**Figure 2:**
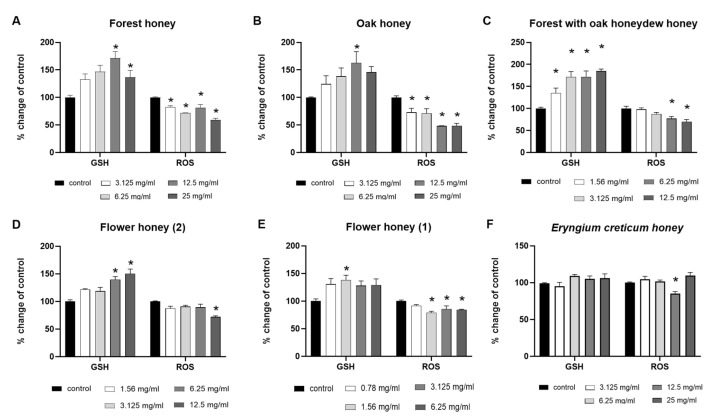
The effects of non-cytotoxic concentrations (0.78 mg/mL–25 mg/mL) of (**A**) Forest, (**B**) Oak, (**C**) Forest with oak honeydew, (**D**) Flower (2), (**E**) Flower (1), and (**F**) Eryngium creticum honey samples on the intracellular GSH and ROS levels of RAW264.7 macrophages. *: *p* < 0.05, statistically significant difference as compared to the control.

After determining the effects on cell proliferation, non-cytotoxic concentrations (0.78 mg/mL–25 mg/mL) of the honey samples were administered to RAW264.7 macrophages to examine their impacts on the intracellular GSH and ROS levels using flow cytometry. According to our findings (Figure 2), most honey samples enhanced GSH levels and reduced ROS levels. To be more specific, the Forest honey sample (Figure 2A) exhibited an increase in GSH levels at highest tested concentrations (12.5 and 25 mg/mL), along with a decrease in ROS levels after cells treatment with all tested concentrations, in comparison with the control. Oak honey sample (Figure 2B) showed an increase in GSH levels at 12.5 mg/mL, as well as a reduction in ROS levels at all tested concentrations, as compared to the control. Furthermore, the Forest with oak honeydew honey sample (Figure 2C) showed a statistically significant increase in GSH levels at all tested concentrations, as well as a reduction in ROS levels as a result of the addition of 6.25 and 12.5 mg/mL honey, as compared to the control. Moreover, Flower (2) honey sample (Figure 2D) showed an increase in GSH levels at highest tested concentrations, as well as a statistically significant decrease in ROS levels at highest tested concentration (12.5 mg/mL), while the Flower (1) honey sample (Figure 2E) exhibited a decrease in ROS levels after treatment, in comparison with the control. Finally, the *Eryngium creticum* honey sample (Figure 2F) showed an isolated effect as it reduced ROS levels to 12.5 mg/mL, compared to the control.

### 2.4. Determination of the Effects on Total Antioxidant Capacity (TAC), Thiobarbituric Acid Reactive Substances (TBARS), and Protein Carbonyls (PCARBS) Levels

**Figure 3:**
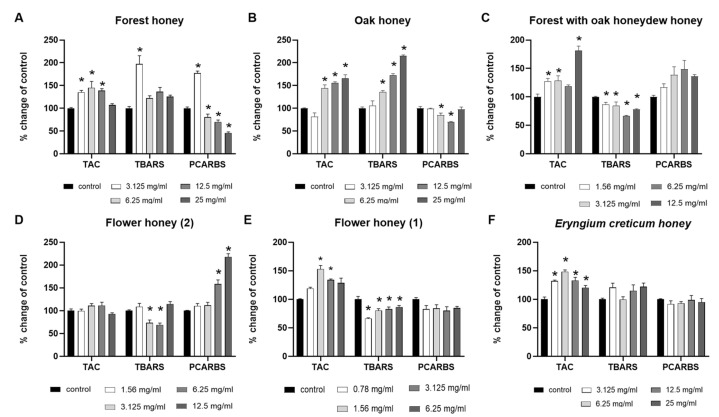
The effects of non-cytotoxic concentrations (0.78 mg/mL–25 mg/mL) of (**A**) Forest, (**B**) Oak, (**C**) Forest with oak honeydew, (**D**) Flower (2), (**E**) Flower (1), and (**F**) Eryngium creticum honey samples on TAC, TBARS, and PCARBS levels of RAW264.7 macrophages. *: *p* < 0.05, statistically significant difference as compared to the control.

Non-cytotoxic concentrations of the honey samples were administered to RAW264.7 macrophages to investigate their impacts on TAC, TBARS, and PCARBS levels by spectrophotometry. Based on our results (Figure 3), most honey samples augmented TAC levels and decreased TBARS or PCARBS levels. More elaborately, the Forest honey sample (Figure 3A) exhibited an increase in TAC levels in all tested concentrations except for the highest, in comparison with the control. Interestingly, a biphasic response phenomenon was observed in PCARBS levels, characterized by a statistically significant decrease, as compared to the control. Although, on lowest tested concentration, there emerged a vast increase in PCARBS levels along with the same response on TBARS levels. The Oak honey sample (Figure 3B) revealed a statistically significant increase in TAC levels at highest tested concentrations (6.25, 12.5, and 25 mg/mL), as well as in TBARS levels at the same concentrations, along with a reduction in PCARBS levels at 6.25 and 12.5 mg/mL, as compared to the control. Furthermore, the Forest with oak honeydew honey sample (Figure 3C) showed an increase in TAC levels, as well as a statistically significant decrease in TBARS levels at all tested concentrations, in comparison with the control. Flower (2) honey sample (Figure 3D) showed a decrease in TBARS levels at 3.125 mg/mL and 6.25 mg/mL, as well as a statistically significant increase in PCARBS levels at 6.25 mg/mL and 12.5 mg/mL, compared to the control. Moreover, the Flower (1) honey sample (Figure 3E) revealed an increase in TAC levels after the addition of 1.56 mg/mL and 3.125 mg/mL honey, accompanied by a decrease in TBARS levels at all tested concentrations, as compared to the control. Finally, the *Eryngium creticum* honey sample (Figure 3F) showed an increase in TAC levels resulting by the addition of all tested honey concentrations, as compared to the control.

## 3. Discussion

The objective of the present study was to evaluate the impact of six raw honey samples, sourced from Pindos Mountain range in Greece, on cellular redox homeostasis. Although the in vivo antioxidant efficacy of luteolin, chrysin, and quercetin, flavonoids present in honey is still unclear, earlier research have demonstrated that they can act as ROS scavengers because of their aromatic structure, hydroxyl groups, and conjugated system [36,37,38]. Indeed, various dietary compounds, extracted from natural sources, such as fruits, vegetables, coffee, and honey have been recognized for their health benefits [32,39].

Herein, the six raw honey samples were initially examined for their polyphenolic content, and, among them, the Forest honey sample exhibited the highest. The term “Forest honey” is used to describe honey produced by nectar and/or honeydew from plants grown in altitudes above 700 m, where the biodiversity is completely different from the lowland area, due to the climatic conditions in mountainous and semi-mountainous areas [40,41]. Previous investigations have indicated that raw honey sourced from Olympus Mountain in Greece has a polyphenolic content ranging from 59 to 78 mg GAE/100 g of honey [42], significantly lower than that of the raw honey samples of our study. Manuka honey, regarded as one of the world’s most premium honey, exhibits significant variations in TPC, ranging from 71 mg GAE/100 g [42], to 492 mg GAE/100 g honey [43]. Research studies on Greek honey samples have demonstrated that oak honey contains significantly higher levels of phenolic compounds compared to other monofloral and flower honey samples [35,44]. Even though the Oak honey does not rank highest in the current study, its phenolic content remains substantial.

Following the characterization of the bioactive content, the redox-related effects of the six raw honey samples were examined in RAW264.7 macrophages. Macrophages are key components of the innate immune system, known for their ability to detect and destroy pathogens, thus, regulating the immune responses [45]. Previous studies have assessed the capacity of honey to induce apoptosis and inhibit cell proliferation [9,46,47,48,49]. However, in this study, we selected non-cytotoxic concentrations of the honey samples to assess their effects on the redox equilibrium of RAW264.7 macrophages.

According to our findings, the Forest with oak honeydew and Flower (1) honey samples exerted beneficial effects on cellular redox homeostasis following the same pattern. To be more specific, they intensified the antioxidant defense mechanisms and lessened ROS, hence preventing lipid peroxidation. Furthermore, the Forest honey sample had positive impacts on cellular redox state. More elaborately, it enhanced the antioxidant defense mechanisms and reduced ROS, therefore, protecting against protein carbonylation. Moreover, the *Eryngium creticum* honey sample had positive effects on cellular redox homeostasis by increasing TAC. The case was different for the Oak honey sample, which caused dual effects on cellular redox state. On the one hand, it enhanced the antioxidant defense mechanisms and diminished ROS, thus, protecting against protein carbonylation; on the other hand, it induced lipid peroxidation. The Flower (2) honey sample also caused dual effects on cellular redox state, resulting by the increase in GSH and the induce in ROS and TBARS, parallel to an increase in PCARBS.

Honey possesses strong antioxidant properties due to its polyphenol and flavonoid content. Polyphenols (e.g., apigenin, quercetin, and kaempferol) may exert their biological activities by modulating the Nrf2 and NF-κB signaling pathways [50,51,52,53]. The Nrf2 signaling pathway is activated under oxidative stress conditions to regulate the expression of several antioxidants and detoxifying genes. Honey contains various bioactive compounds which can stimulate the pathway and promote the translocation of Nrf2 from the cytoplasm to the nucleus, enhancing the transcription of cytoprotective genes, such as SOD, CAT, and GPx [54]. Previous investigations have examined the effects of Manuka honey on RAW264.7 cells, indicating that it exerts its antioxidant activities by reducing ROS generation, protecting against lipid, protein, and DNA damage and upregulating the expression of Nrf2, SOD, CAT, and HO-1 genes [55]. Furthermore, Manuka honey prevents from oxidative damage and maintains mitochondrial functionality through the activation of the AMPK/Nrf2 signaling pathway, upregulating the expression of antioxidant genes, such as SOD and CAT [56].

The most efficacious raw honey samples, as regards their impact on redox homeostasis, were the Forest and Forest with oak honeydew. Noteworthy, the Forest with oak honeydew honey sample also exhibited the highest total phenolic content. It is worth mentioning that honey enriched with honeydew has a darker color and a different composition than nectar honey, as it has elevated polyphenolic content, including compounds such as myricetin and pinobanksin, which are exclusive to this type [57,58,59]. A prior study in HepG2 cells has revealed that similar raw honey samples to those of the study induce a different cell response, reducing both GSH and ROS levels [60].

Honey is well-known for its health-promoting properties; however, research of its mechanism of action is scarce due to its vast diversity, owing mostly to its plant type. Beehive’s location is the most important factor affecting honey’s properties since different locations means different beehive environment, soil composition and climatic conditions [61,62,63]. It is well-established that honeys with same floral source, harvested by beehives in different areas often differ when it comes to its composition [64,65]. This argument is confirmed in the present study, considering that the two Flower honey samples had different responses to the redox markers. Finally, the bioactive composition of the final product is also influenced by the beekeepers’ technique as well as the storage conditions [66].

Despite the fact that the exact antioxidant mechanism of honey is unclear, some evidence indicates its involvement in the Nrf2 transcription factor pathway, a common antioxidant response activated under low levels of oxidative stress. Previous research has shown that honey can stimulate the activation of Nrf2 by promoting its translocation to the nucleus, where it induces the expression of targeted antioxidant and detoxifying genes, such as SOD, CAT, and GPX [56,67].

Although honey is known for its beneficial properties, the information about its molecular mechanism of action is still limited due to its great diversity. Factors such as location, climatic conditions, soil composition, and the overall environment around beehives influence the honey produced and intensify the differences even between honey of the same species [62]. Global literature suggests that honey with different floral sources have different biochemical profiles [63]. In conclusion, the current study highlights raw honey’s strong antioxidant capacity, confirming its promising role in redox homeostasis. Despite the positive results, further studies are needed to understand the mechanism of action of honey as it is a very complex mixture with high interspecies variability. Given the complexity of responses observed at different concentrations, further research on cell-based systems and in more advanced biological models, it is suggested to clarify the antioxidant mechanism of action.

## 4. Materials and Methods

### 4.1. Sample Preparation

Six raw honey samples, originated from multiple sites along the Pindos Mountain range in Greece, were obtained from small-scale producers. Each honey sample was diluted in deionized water (dH_2_O) at a 1:1 *w/v* ratio and heated for 5 min at 35–40 °C. Then, the honey samples were left for 15 min at room temperature (RT).

### 4.2. Total Phenolic Content (TPC)

To estimate TPC we used Folin–Ciocalteu (FC) reagent. In a plastic tube of 2 mL volume, we added 1 mL of dH_2_O, 20 μL of each honey sample, and 100 μL of FC, followed by a 4 min incubation. We then added 280 μL of a sodium carbonate solution 25% (*w/v*) and 600 μL of dH_2_O. The samples were incubated in the dark for 55 min at RT and the optical density was measured at 765 nm using a UV/Vis spectrophotometer (VWR, UV/VIS 1600PC, VWR International, Radnor, PA, USA). Results were expressed as mg of gallic acid equivalent (GAE)/100 g of honey. We created a standard curve of Gallic acid (GA) using a wide range of sequential concentrations from 25 to 500 µg/mL [68]. We conducted the experiment on three different occasions and in triplicates.

### 4.3. Cell Cultures

RAW264.7 is a well-established cell line used for assessing immune system, metabolic and phagocytic functions [55]. We purchased them from the American Type Culture Collection (ATCC, Manassas, VA, USA). The cells were cultured in disposable plastic cell culture flasks (75 cm^2^) using Gibco Dulbecco’s modified Eagle’s medium (DMEM), containing 10% (*v/v*) fetal bovine serum (FBS), 2 mM L-glutamine, 100 U/mL penicillin, and 100 U/mL streptomycin in 5% carbon dioxide (CO_2_), 80–95% humidity and 37 °C to reach 70–80% confluency. Cells were passaged during their exponential growth phase. The culture medium has been routinely changed, and we detached them by using cell scraper. Using RT-PCR, we tested them for mycoplasma contamination, while we looked over daily microscopically on an inverted microscope (OCL 251, Kern Optics Balingen, Germany,), looking for unsolicited morphology and/or growth alterations.

### 4.4. Cell Viability Assay

We used a commercially available kit (by R&D Systems, Inc., Minneapolis, MN, USA) to identify honey samples effect on cell viability. Pursuant to the assay principle, normally cells can use their dehydrogenase activity, to transform the tetrazolium salt (XTT), to a (water-soluble) formazan product. More elaborately, the RAW264.7 macrophages were seeded in a 96 well-plate at a density of 10^4^ cells/well and cultured in complete culture medium for 24 h, followed by the addition of a wide range of sequential concentrations from 0.78 to 50 mg/mL, in culture medium in the absence of FBS. After another 24 h incubation, we added the XTT reagent in total volume of 50 μL in each well plate (49 µL XTT-labeling reagent and 1 µL XTT activator), followed by the last incubation for a duration of 4 h. We used a plate reader (ENZO, Absorbance 96 Plate Reader, Byonoy GmbH, Hamburg, Germany) to measure the optical density (OD) at 450 nm and the reference wavelength at 630 nm. The untreated cells served as the control group, while all tested honey samples were measured in the absence of cells, (negative control) and subtracted from the corresponding values including cells. The percentage change in cell viability was calculated as follows: % change in control = (ODsample/ODcontrol) × 100, where ODcontrol and ODsample represent the optical density of control and the test compound. We conducted the experiment on three different occasions and in triplicates.

### 4.5. Flow Cytometry

We used flow cytometric analysis to evaluate alterations on GSH and ROS levels. More specifically, the RAW264.7 macrophages were seeded at a density of 10^5^ cells/well and cultured in complete medium, for 24 h. Then, different concentrations of the honey samples (after XTT analysis), diluted in Gibco DMEM without the addition of FBS, were added, and the cells were incubated for 24 h. Afterwards, cells were trypsinized, centrifuged (1400× *g*, 4 min, 4 °C), resuspended in phosphate-buffered saline (PBS), and then centrifuged again. The supernatant was discarded, and Thiol Green (125 μM) was added to measure intracellular GSH levels followed by a half hour incubation at 37 °C. Samples were then centrifuged under the conditions described above and resuspended in PBS. For intracellular ROS levels evaluation, we followed the instructions described for GSH evaluation, although we used H_2_DCFDA (10 μM). Both dyes were diluted in PBS. The analysis was performed on a 10^4^ cells/sample at a rate of approximately 300 events/second on a logarithmic scale (on the (FACSCalibur, BD Biosciences, Franklin Lakes, NJ, USA). The BD Cell Quest Pro software v 6.0 (BD Biosciences) was used to analyze the data. We conducted the experiment on three different occasions and in triplicates [60,69].

### 4.6. Spectrophotometry

The spectrophotometric analysis was performed for the examination of the effects on TAC, TBARS, and PCARBS levels. More elaborately, the RAW264.7 macrophages were seeded in disposable plastic cell culture flasks (75 cm^2^) at a density of 2 × 10^6^ cells/flask and cultured in Gibco DMEM, supplemented with complete culture medium, for 24 h. Then, different concentrations of the honey samples (0.78 mg/mL–25 mg/mL), diluted in Gibco DMEM without the addition of FBS, were added, and the cells were incubated for 24 h. After incubation, cells were detached by using cell scraper in PBS containing protease inhibitors (Complete™ mini protease inhibitors, Roche Applied Science) and lysed by periodical ultrasonication. The Bradford assay was used for estimating the total protein concentration using a bovine serum albumin standard curve [70].

For TAC levels evaluation, a volume of cell lysate that contains 40 μg of total protein was added to 10 mM of phosphate buffer (pH 7.4) in a total volume of 500 μL. In total, 500 μL of a DPPH^•^ solution (0.1 mM) diluted in methanol was added, and the samples were then incubated in the dark at RT for 55 min. After this incubation, samples were centrifuged for 3 min, at 12,000× *g*, at room temperature. Optical density was measured at 520 nm using a UV/Vis spectrophotometer (VWR, UV/VIS 1600PC, VWR International, Radnor, PA, USA). Results were calculated after the reduction of DPPH^•^ to DPPH:H (2,2-diphenyl-1-picrylhydrazine) [71,72].

For TBARS determination, a modified version of a protocol described by of Keles et al. [73] was performed. A volume of cell lysate that contains 80 μg of total protein was added to PBS in a total volume of 500 μL. Then, 200 mM of a pH 7.4 (500 μL) Tris-HCl buffer and 500 μL of 30% w/v trichloroacetic acid (TCA) were added followed by a 9 min incubation at room temperature. A total of 1 mL of 2 M sodium sulfate (Na_2_SO_4_) and 55 mM thiobarbituric acid (TBA) were added, followed by a 45 min incubation at 95 °C. The samples then cooled in ice for approximately 4 min. A total of 1 mL of 70% *w/v* TCA was added, and 1 mL of it was transferred in a 1.5 mL tube and centrifuged for 5 min in 11,000× *g* at room temperature. Optical density was measured at 530 nm using a UV/Vis spectrophotometer (VWR, UV/VIS 1600PC, VWR International, Radnor, PA, USA). Results were calculated based on the extinction co-efficient of malondialdehyde (MDA) [74].

An assay that was previously described by Patsoukis et al. [70] was performed for the evaluation of PCARBS levels. A volume of cell lysate that contains 80 μg of total protein was added to PBS in a total volume of 500 μL. Considering that every sample has its own blank, 0.5 mL of 2.5 N hydrochloric acid (HCl) was added in blanks and 0.5 mL of 10 mM 2.4-dinitrophenylhydrazine (DNPH) in samples. An hour incubation in the dark was followed by centrifugation at 12,000× *g* for 5 min at 5 °C. Then, the supernatant was discarded, and 950 μL 10% *w/v* TCA was added followed by a centrifuge as described above. Supernatant was discarded again, and the pellet was resuspended in 1000 μL of a 1:1 ethanol–ethyl acetate solution followed by centrifugation as described above. This step was repeated another time, followed by the addition of 1000 μL of 5 M (pH 2.3 Urea) and 14 min incubation at 37 °C. Samples were then centrifuged as described at previous steps of the assay. Optical density was measured at 375 nm using a UV/Vis spectrophotometer (VWR, UV/VIS 1600PC, VWR International, Radnor, PA, USA). Results were calculated based on the molar extinction co-efficient of DNPH [75].

### 4.7. Statistical Analysis

For data interpretation, we used one-way analysis of variance (ANOVA), followed by Dunnett’s post-hoc test, to detect statistically significant differences (*p* value ≤ 0.05) between the untreated cells (control group) and the cells treated with the tested concentrations. In the current study, results were presented as mean ± standard error of the mean (SEM) and analyzed by GraphPad Prism (GraphPad Prism version 8.0.1 for Windows, GraphPad Software, Inc., La Jolla, CA, USA).

## Figures and Tables

**Table 1:** The Total Phenolic Content (TPC) of the honey samples expressed as mg gallic acid equivalents (GAE)/100 g honey.

Honey Samples	TPC (mg GAE/100 g Honey)
Forest	129.4
Oak	124
Forest with oak honeydew	115.4
Flower (2)	89.5
Flower (1)	86.1
Eryngium creticum	83.8

## Data Availability

The raw data supporting the conclusions of this article will be made available by the authors on request.
